# An efficient method for the selective isolation of feline herpesvirus 1(FHV-1) in feline calicivirus (FCV) coinfected specimens

**DOI:** 10.1186/s12917-025-04786-w

**Published:** 2025-05-07

**Authors:** Huanqin Zheng, Hong Yue, Baoyan Wang, Xin Yu, Yang Liu, Jiayu Yu, Jianlong Zhang, Kexue Han, Yinuo Han, Hanfeng Su, Hongwei Zhu, Xingxiao Zhang

**Affiliations:** 1 School of Life Sciences, Ludong University, No. 186 Hongqi Middle Rd., Zhifu District, Yantai, 264025 China; 2 Collaborative Innovation Center for the Pet Infectious Diseases and Public Health in the Middle and Lower Stream Regions of the Yellow River, Yantai, 264025 Shandong China; 3 Provincial Engineering Research Center for Pet Animal Vaccines, Yantai, 264025 Shandong China; 4 Jinan Baiming Biopharmaceutical Co., Ltd, Jinan, 250101 Shandong China; 5 College of Veterinary Medicine, Jilin Agricultural University, Changchun, 130118 China

**Keywords:** Coinfection, Feline herpesvirus 1, Feline calicivirus, Virus isolation

## Abstract

**Background:**

Feline herpesvirus 1 (FHV-1) and feline calicivirus (FCV) are the most common viral pathogens of feline respiratory disease and are highly prevalent in cats worldwide. Coinfection with these viruses is frequent in cats with feline respiratory disease complex (FRDC). It is difficult to isolate pure FHV-1 by conventional laboratory cell culture methods from specimens with FRDC, which brings great trouble to the epidemiological investigation of FHV-1.

**Methods:**

FCV polyclonal antibodies were obtained by immunizing rabbits, and the coinfected specimens were neutralized with FCV polyclonal antibodies. Then, virus isolation was performed. After several rounds of neutralization, FHV-1 was finally obtained.

**Results:**

The FCV polyclonal antibody was successfully obtained with neutralizing activities of 1:128, 1:537, and 1:91. After virus neutralization, the FHV-1 was successfully isolated from the coinfected cell culture suspension and confirmed by immunofluorescence and QRT-PCR.

**Conclusion:**

In this study, all FHV-1 present in the coinfection samples were isolated, without any cross-contamination. This method is also theoretically suitable for the isolation and purification of other FCV coinfections or contaminating disease substances.

## Background

Feline respiratory disease complex (FRDC), which is also referred to simply as feline upper respiratory infection (URI), is a common infection in domestic cats and is most prevalent in stressful crowded feline populations and is the second leading cause of euthanasia in shelters after overcrowding [1–3]. Feline herpesvirus 1 (FHV-1) and feline calicivirus (FCV) are the most common viral pathogens of FRDC, and coinfection with these viruses is frequent in cats [4–6]. Clinical signs caused by FHV-1 are rhinitis and conjunctivitis, characterized by sneezing and oculonasal serous discharge, which may be accompanied by hypersalivation with drooling, depression, inappetence, and pyrexia [7–9]. Clinical signs caused by FCV are typically characterized by oral ulcerations with or without mild respiratory and conjunctival signs [10, 11]. Therefore, the majority of cats with FRDC have FCV and FHV-1 coinfection.

The detection of coinfection using molecular methods has been reported from many parts of the world, but isolation of both viruses in cell culture is commonly used to completely characterize the pathogen [12]. Usually, it is difficult to isolate pure FHV-1 by conventional laboratory cell culture methods from coinfection samples with FRDC, which brings great trouble to the epidemiological investigation of FHV-1. Because FCV is characterized by continuous shedding during infection, while FHV-1 has shorter periods of shedding, FCV is isolated more commonly than FHV-1 from cats with FRDC [11, 13–15]. Because most of the samples are coinfecte and FCV produces cytopathic effect (CPE) early, it affects the replication of FHV-1 during cell culture [16–18]. Even plaque purification also has difficulty obtaining pure FHV-1. Moreover, pure FHV-1 contaminated with FCV during routine operation also causes FHV-1 to disappear rapidly after several rounds of multiplication. Therefore, it is difficult to calculate the true prevalence of FHV-1 in clinical samples [5].

Due to the above problems, we tried to apply the virus neutralization method, pretreated the samples, and completely neutralized the FCV after several rounds of treatment, which increased the success rate of FHV-1 isolation, and successfully isolated 4 strains by applying this method. This method is also theoretically suitable for the separation and purification of FCV mixed infections or contaminated specimens.

## Results

### Antisera preparation against FCV

FCV polyclonal antibodies were successfully obtained by immunology of rabbits by laboratory-isolated FCV virus. The serum of the collected rabbits was tested for FCV neutralizing antibody titer, and the results showed that FCV antibodies were produced on the 28 th day after immunization, with antibody titers of 1:8, 1:16, and 1:4. The serum of the three rabbits collected on the 60 th day produced the highest FCV antibody titer, with antibody titers of 1:128, 1:537, and 1:91, respectively.

### Coinfection sample isolation and identification

**Fig. 1:**
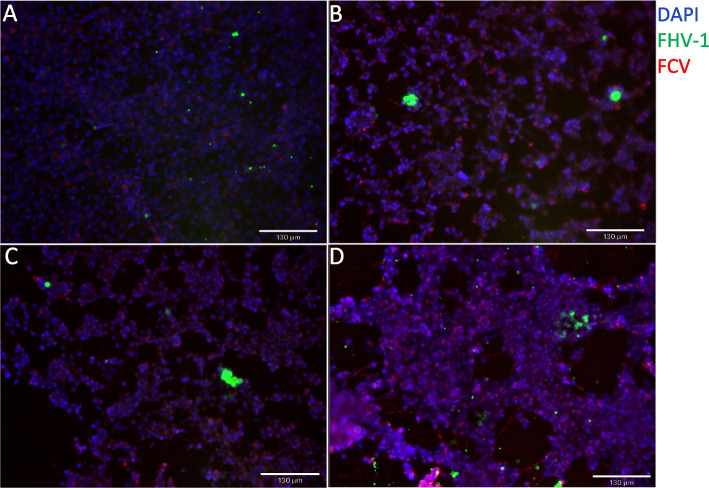
Immunofluorescence of FHV-1 and FCV from the coinfected samples. **A**-**D** show different hours post infection (hpi). **A** were taken at 6 hpi. **B** were taken at 12 hpi. **C** were taken at 18 hpi, and **D** were taken at 24 hpi. DAPI showed a blue fluorescence signal and was used to detect the cell nucleus. FHV-1 showed a green fluorescence signal, and FCV showed a red fluorescence signal

CPE was observed in CRFK cells infected with virus for 6 h-24 h. When the first virus isolation was performed, after 6 h, the cells became round and showed karyopyknosis, wizened weakening, and aggregation in comparison to the control cells, which is consistent with the CPE of FCV, and gradually dominated with increasing infection time. FHV-1 can detect only a few fluorescence signals (Figure 1).

### Isolation and identification after virus neutralization

**Fig. 2:**
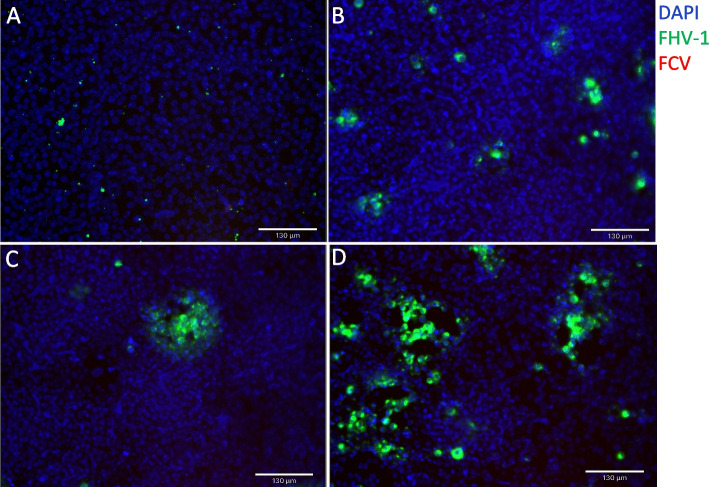
Immunofluorescence of FHV-1 and FCV after neutralization. **A**-**D** show different hours post infection (hpi). **A** were taken at 6 hpi. **B** were taken at 12 hpi. **C** were taken at 18 hpi, and **D** were taken at 24 hpi. DAPI showed a blue fluorescence signal and was used to detect the cell nucleus. FHV-1 showed a green fluorescence signal, and FCV showed a red fluorescence signal

After virus neutralization, at 6–24 h post-inoculation, CRFK cell cultures showed a distinct CPE of FHV-1, characterized by cell rounding, pyknosis, Fleece-Pulling’ to the thyrsoid and degeneration of the cell monolayer. With increasing infection time, FHV-1 expression also gradually increased (Figure 2).

### Quantitative real-time PCR assay

**Fig. 3:**
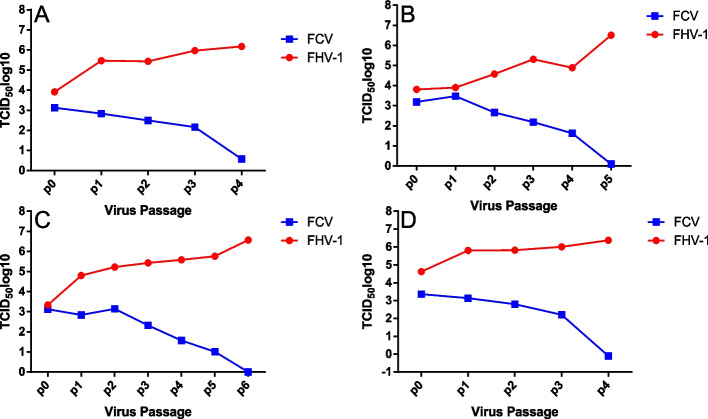
Growth kinetics of FHV-1 and FCV from the coinfected samples. **A**-**D** show different samples. In this figure, 0 copies indicates no Ct data in QRT-PCR

As the threshold cycle (Ct) values showed near perfect linear regression with the copies [19–21], the Ct values were extrapolated into virus copies from the standard curve to monitor the virus content. With increasing neutralization time, the content of FHV-1 gradually increased, while FCV gradually decreased. FHV-1 was successfully isolated from the coinfected cell culture suspension and was confirmed by QRT-PCR (Figure 3).

## Discussion

FHV-1 and FCV are the main viral pathogens of FRDC, and coinfection of FCV and FHV-1 often occurs in clinical practice. In most of the clinical materials obtained from the coinfected animals, only one virus is isolated, whereas the others get eliminated in subsequent passages [21]. Because FCV is continuously shed and produces CPE earlier, it is difficult to isolate FHV-1 from coinfection samples, which brings certain difficulties to clinical diagnosis and molecular epidemiological investigation of viruses. Previous studies also report low isolation rates of FHV-1 from cats [11], so the isolation of FHV-1 in coinfected samples is essential.

Before this study, in the samples with FCV and FHV-1 coinfection, people usually thought that FHV-1 may not be expressed due to FCV inhibition in the later stage. This study found that FHV-1 is also expressed in coinfected samples, possibly due to the premature production of CPE by FCV, which limits the expression of FHV-1 in host cells, and the specific reasons need to be further explored.

QRT-PCR was used for virus quantification in this study. Compared with conventional PCR, QRT-PCR has higher sensitivity and is more conducive to the detection of virus content in clinical samples. If necessary, the method of expressing viral content in CT values can also be extrapolated to other viruses because CT values and viral content are linearly related, which has been reported in other articles [20, 21].

The four clinical samples we obtained were all FCV coinfected with FHV-1, and we tried to obtain pure FHV-1 by plaque purification, but this method ended in failure after several rounds of testing. The reason may be that when the two viruses proliferated in cells at the same time, the cytopathic effect of the slower proliferating FHV-1 is suppressed by the relatively faster proliferating FCV, or that CRFK cells co-infected with the two would show similar CPE, making it difficult to distinguish the two [15]. What’s more, during the process of selecting FHV-1 plaques, it is inevitable to carry some FCV plaques, which showed growth advantages during the later inoculation process, thus inhibiting the replication of FHV-1, and ultimately causing the failure of plaque purification. Methods for obtaining the virus of interest by virus neutralization have been previously reported. For example, the purified avian influenza virus was successfully obtained from avian influenza and Newcastle disease co-infected samples by virus neutralization [21, 22]. Since FCV has high genetic variability and genetic diversity, it is difficult to find suitable monoclonal antibodies [23], so we chose to produce polyclonal antibodies against FCV.

Usually, FCV is often unknown in co-infection samples. In order to improve the neutralization effect, FCV in samples can be purified first, and then used as an antigen to prepare FCV polyclonal antibodies. In this way, polyclonal antibodies with high specificity can be obtained, which is theoretically applicable to any FCV co-infection samples but this method need more time [24]. In order to obtain our target virus FHV-1 quickly, the original FCV in our laboratory was used for immunization. By immunizing rabbits, we successfully obtained FCV polyclonal antibodies with neutralizing activity. The different titers of neutralizing antibodies produced after immunizing animals may be caused by individual differences in animals. To neutralize FCV more effectively in this research, the serum with the highest neutralizing antibody was selected to neutralize the coinfected samples, and they were mixed 1:1, through 4 to 6 around of neutralization, pure FHV-1 is obtained. In the neutralization process, each neutralization is carried out in strict accordance with the steps, and the samples after each neutralization are verified by IFA and QRT-PCR, in order to avoid the procedural errors and variability that could be caused by repeated neutralization processes. Optimization of antibody working concentration is the prerequisite and key to successful virus isolation, there may have a better ratio to shorten the time of virus purification, but this condition needs to be further explored. The limitation of the method descried in this study is that the polyclonal antibodies prepared targeting one laboratorial FCV strain was not enough for all field isolates, but this method could facilitate and improve the success probability of FHV-1 isolating. This method has important practical significance especially when the sample is precious.

## Conclusion

In this study, we obtained FCV polyclonal antibodies by immunizing rabbits with FCV and then successfully obtained pure FHV-1 from coinfected samples by virus neutralization. This study will provide a useful reference for the isolation and purification of FCV coinfection or contaminated specimens.

## Materials and methods

### Specimen, cell and virus

The samples consisted of conjunctival, nasal and, occasionally, oral and oropharyngeal swabs collected from cats with clinical signs of FRDC from one pet hospital in the study. The Crandell feline kidney cell line (CRFK) was purchased from Procell Life Science & Technology Co., Ltd. The FCV virus was isolated and stored in our laboratory.

### Inoculum treatment and virus isolation

Nose, pharynx and conjunctiva samples were obtained by gently rubbing a sterile cotton swab. Swabs were placed in a solution of 1.0 mL phosphate-buffered saline (PBS, pH 7.2), freeze thawed 3 times, and centrifuged at 5000 rpm for 15 mins. The supernatant was filtered with disposable 0.22 μm syringe filters. The CRFK cells were cultured to 80% confluence in Dulbecco's modified Eagle's medium (DMEM) supplemented with 10% fetal bovine serum (FBS) and 1% penicillin‒streptomycin solution at 37 °C with 5% CO_2_. The treated samples were diluted 1:1000 with DMEM without penicillin‒streptomycin solution and FBS and then inoculated into CRFK cells at 37 °C for 90 min. DMEM containing 2% FBS and 1% penicillin‒streptomycin solution was then incubated with 5% CO_2_ at 37 °C. The infected cultures were incubated at 37 °C for 3–4 days, and the presence or absence of cytopathic effect (CPE) was recorded. The infected cells and media in the flask were then frozen at −80°C and thawed at room temperature for 3 cycles. The supernatant was removed from the flask, placed in a conical tube and centrifuged at 3000 rpm for 5 min. The supernatants were collected for PCR detection to isolate the virus.

### FHV-1 plaque purification

The CRFK cells were cultured to 80% confluence in 6-well plates. Samples were diluted a series of ten-fold in DMEM without penicillin‒streptomycin solution and FBS to make 10^−1^ to 10^−8^ dilutions, and 10^−3^ to 10^−8^’s dilutions were inoculated into CRFK cells at 37 °C for 90 min. Then CRFK cells were washed with PBS and overlaid with DMEM containing 1 % low-melting-point agarose (Lonza, 50101) and 2 % FBS, and incubated for 72 h at 37℃ under 5 % CO_2_. A sterile pipette tip was used carefully to pick a CPE of FHV-1 plaque. The agarose containing the plaque was transferred into a tube with 1 mL DMEM. The plaques were frozen at −80°C and thawed at room temperature for 3 cycles, and then were inoculated into CRFK cells. The same operation is repeated three times, and the last plaque with DMEM were centrifuged at 3000 rpm for 5 min, and the supernatant was taken for virus identification.

### Selective FHV-1 isolation

To make polyclonal antisera against FCV, 3 healthy 2-month-old New Zealand rabbits obtained from Qingdao Kangda Aibo Biotechnology Co., Ltd (Qingdao, China) were immunized with the purified FCV virus. A protein concentration was performed on the purified FCV virus using a Pierce(tm) BCA Protein Assay Kit (Thermo Fisher, 23227-500 mL) according to the manufacturer’s instructions and demonstrated a concentration of 78.55 μg/mL. The FCV virus was concentrated to 800 μg/mL with Macrosep Advance centrifugal devices (PALL, MAP100 C38). In each rabbit, on day 0, 1 mg of concentrated FCV virus emulsified 1:1 in complete Freund’s adjuvant (Sigma‒Aldrich, F5881) was given by the intramuscular injection route divided into ten sites. The second and third immunizations (identical protocol) were administered with incomplete Freund’s adjuvant (Sigma‒Aldrich, F5506) on days 15 and 30. Blood was obtained from the rabbits at baseline and on days 14, 21, 28, 37, and 60 from the central ear artery. Serum was harvested and banked at −20°C until further preparation and characterization.

Then, 100 μL of the coinfected cell culture supernatant was mixed with 100 μL of serum, and virus/serum mixtures were incubated at 37 °C for 1–2 hours. Then, CRFK cells at 70–80% confluency in 6-well cell culture plates were washed with PBS, followed by transfer of the virus/serum mixture. The CRFK cells were incubated at 37 °C for 1 h, DMEM containing 2% FBS and 1% penicillin‒streptomycin solution was added, and the plates were incubated at 37 °C for 3–4 days. The supernatant was then collected, and the virus was isolated and identified.

### Immunofluorescence staining

Briefly, 6 h, 12 h, 18 h and 24 h after infection, a monolayer of cells cultured on cover slips was fixed with 4% paraformaldehyde for 15 min. The samples were incubated with mouse anti- FHV-1 antibody (EastCoast Bio, HM550) and our laboratory-prepared FCV polyclonal antibody at 37 °C for 30 min in a humid box and then with goat anti-rabbit IgG (Proteintech, SA00013-4) and goat anti-mouse IgG (Abcam, ab150113) for 30 min at 37 °C. The nuclei were stained with DAPI (Sigma‒Aldrich, D9542), and slides were mounted in Antifade Mounting Medium (Beyotime Biotechnology, P0126). Images were acquired using a Nikon Eclipse Ni-U fluorescence microscope with NIS-Elements. Blank CRFK cells were analyzed as a negative control.

### Virus quantitation

The TaqMan chemistry-based real-time (QRT-PCR) assay was used for the quantification of FHV-1 and FCV. The primers and probes specific for FHV-1 and FCV were designed in this study (Table 1). Total DNA/RNA was extracted as described in the previous section. Using RNA was used as a template, and single-stranded cDNAs were generated with a RevertAid First Strand cDNA Synthesis Kit (Thermo, K16225) according to the manufacturer’s directions. AceQ qPCR Probe Master Mix (Vazyme, Q112-02) was used for assembling the serotype-specific TaqMan QRT-PCR. QRT-PCR was performed in a 7500 Cycler (Applied Biosystems, USA) under the following conditions: initial denaturation at 95 °C for 15 min, followed by 40 cycles of denaturation at 95 °C for 10 s and annealing at 60 °C for 35 s.

**Table 1:** Details of QRT-PCR primers and probes used in the study

Name of the primer/probe	Primer/probe sequence
FHV-probe	TATGTGCTTTACGGTCGCCT
qFHV-F	ATGAGACTTTGTGATCCTAAACGGG
qFHV-R	TCAAACCCAGTTCATCGTCTGTTAG
FCV-probe	TCGGTGTTTGATTTGGCCTG
qFCV-F	ACTACCCGCCAATCAACATGT
qFCV-R	AGTCAATGTCAGGTGTCGGC

## Data Availability

The datasets used and/or analyzed during the current study are available from the corresponding author upon reasonable request.

## Abbreviations

FHV-1: Feline herpesvirus 1
FCV: Feline calicivirus
CPE: Cytopathic effect
PCR: Polymerase chain reaction
QRT-PCR: Quantitative real-time polymerase chain reaction

## References

[1] Nguyen D, Barrs VR, Kelman M, Ward MP. Feline upper respiratory tract infection and disease in Australia. J Feline Med Surg. 2019;21(10):973–8. 30465616 10.1177/1098612X18813248 PMC11132241

[2] Di Martino B, Di Francesco CE, Meridiani I, Marsilio F. Etiological investigation of multiple respiratory infections in cats. New Microbiol. 2007;30(4):455–61. 18080682

[3] Bannasch MJ, Foley JE. Epidemiologic evaluation of multiple respiratory pathogens in cats in animal shelters. J Feline Med Surg. 2005;7(2):109–19. 15771947 10.1016/j.jfms.2004.07.004 PMC10822251

[4] Cohn LA. Feline respiratory disease complex. Vet Clin North Am Small Anim Pract. 2011;41(6):1273–89. 22041216 10.1016/j.cvsm.2011.07.006

[5] Helps CR, Lait P, Damhuis A, Björnehammar U, Bolta D, Brovida C, Chabanne L, Egberink H, Ferrand G, Fontbonne A, et al. Factors associated with upper respiratory tract disease caused by feline herpesvirus, feline calicivirus, Chlamydophila felis and Bordetella bronchiseptica in cats: experience from 218 European catteries. Vet Record. 2005;156(21):669–73. 15908495 10.1136/vr.156.21.669

[6] Hoskins JD. Feline respiratory diseases. Vet Clin North Am Small Anim Pract. 1999;29(4):945–58 vii. 10390794 10.1016/s0195-5616(99)50083-1

[7] Monne Rodriguez JM, Leeming G, Köhler K, Kipar A. Feline herpesvirus pneumonia: investigations into the pathogenesis. Vet Pathol. 2017;54(6):922–32. 28812530 10.1177/0300985817720982

[8] Gaskell R, Willoughby K. Herpesviruses of carnivores. Vet Microbiol. 1999;69(1–2):73–88. 10515274 10.1016/s0378-1135(99)00092-9

[9] Gaskell R, Dawson S, Radford A, Thiry E. Feline herpesvirus. Vet Res. 2007;38(2):337–54. 17296160 10.1051/vetres:2006063

[10] Radford AD, Coyne KP, Dawson S, Porter CJ, Gaskell RM. Feline calicivirus. Vet Res. 2007;38(2):319–35. 17296159 10.1051/vetres:2006056

[11] Holst BS, Berndtsson LT, Englund L. Isolation of feline herpesvirus-1 and feline calicivirus from healthy cats in Swedish breeding catteries. J Feline Med Surg. 2005;7(6):325–31. 15914057 10.1016/j.jfms.2005.03.002 PMC10822419

[12] Litster A, Wu CC, Leutenegger CM. Detection of feline upper respiratory tract disease pathogens using a commercially available real-time PCR test. Vet J. 2015;206(2):149–53. 26324635 10.1016/j.tvjl.2015.08.001

[13] Field HJ, Biswas S, Mohammad IT. Herpesvirus latency and therapy–from a veterinary perspective. Antiv Res. 2006;71(2–3):127–33. 10.1016/j.antiviral.2006.03.018 16843537

[14] Harbour DA, Howard PE, Gaskell RM. Isolation of feline calicivirus and feline herpesvirus from domestic cats 1980 to 1989. Vet Record. 1991;128(4):77–80. 1850183 10.1136/vr.128.4.77

[15] Binns SH, Dawson S, Speakman AJ, Cuevas LE, Hart CA, Gaskell CJ, Morgan KL, Gaskell RM. A study of feline upper respiratory tract disease with reference to prevalence and risk factors for infection with feline calicivirus and feline herpesvirus. J Feline Med Surg. 2000;2(3):123–33. 11716607 10.1053/jfms.2000.0084 PMC10829116

[16] Monne Rodriguez J, Köhler K, Kipar A. Calicivirus co-infections in herpesvirus pneumonia in kittens. Vet J. 2018;236:1–3. 29871741 10.1016/j.tvjl.2018.04.004

[17] Sun H, Li Y, Jiao W, Liu C, Liu X, Wang H, Hua F, Dong J, Fan S, Yu Z, et al. Isolation and identification of feline herpesvirus type 1 from a South China tiger in China. Viruses. 2014;6(3):1004–14. 24590411 10.3390/v6031004 PMC3970135

[18] Guo H, Miao Q, Zhu J, Yang Z, Liu G. Isolation and molecular characterization of a virulent systemic feline calicivirus isolated in China. Infect Genet Evol. 2018;65:425–9. 30176370 10.1016/j.meegid.2018.08.029

[19] Horsington J, Zhang Z. Analysis of foot-and-mouth disease virus replication using strand-specific quantitative RT-PCR. J Virol Methods. 2007;144(1–2):149–55. 17561277 10.1016/j.jviromet.2007.05.002

[20] Sachs LA, Schnurr D, Yagi S, Lachowicz-Scroggins ME, Widdicombe JH. Quantitative real-time PCR for rhinovirus, and its use in determining the relationship between TCID50 and the number of viral particles. J Virol Methods. 2011;171(1):212–8. 21070809 10.1016/j.jviromet.2010.10.027

[21] Mahajan S, Sharma GK, Subramaniam S, Biswal JK, Pattnaik B. Selective isolation of foot-and-mouth disease virus from coinfected samples containing more than one serotype. Braz J Microbiol. 2021;52(4):2447–54. 34478107 10.1007/s42770-021-00604-1 PMC8578238

[22] El Zowalaty ME, Chander Y, Redig PT. Abd El Latif HK, El Sayed MA, Goyal SM: Selective isolation of Avian influenza virus (AIV) from cloacal samples containing AIV and Newcastle disease virus. J Vet Diagn Invest. 2011;23(2):330–2. 21398457 10.1177/104063871102300222

[23] Pesavento PA, Chang KO, Parker JS. Molecular virology of feline calicivirus. Vet Clin North Am Small Anim Pract. 2008;38(4):775–86 vii. 18501277 10.1016/j.cvsm.2008.03.002

[24] Xu X, Zheng Y, Liu D, Ma B, Liu J, Qu L. Isolation and Identification of a Feline Herpesvirus-1 Strain. Acta Veterinaria et Zootechnica Sinica. 2023;54(04):1713–20.

